# The Amick Cure

**Published:** 1893-02

**Authors:** 


### THE AMICK CURE.

Our readers have heard about the Amick Cure. To enlighten them further, we reproduce the following editorial from the *St. Louis Clinique*:

"Repeatedly we have been asked about the 'Amick Cure' for consumption. Each inquiry suggests a shameless act upon the part of a well-known and once esteemed professor of ophthalmology in Cincinnati. Here is the history as it has come to us. The discoverer (?) announces that he has found a remedy for the cure of tuberculosis—possibly a modification of Shirley and Gibbes' formula, or at least some disguised alterative. His position gives him a hearing in some of the medical journals. Circulars and 'cases' are next in order. Patients are furnished with medicines costing them about fifteen dollars a month—costing the 'discoverer' much less. Extra inducements are held out to physicians.

"Soon the scheme looks promising from a financial standpoint. A company is formed. It heralds 'a thoroughly reliable treatment and absolute cure for consumption, asthma, chronic bronchitis, tubercular laryngitis, catarrh and hay fever.' No difference about the etiology or pathology, it cures them all. Keeley's nonsense is pure logic compared to this absurdity. No need of condemning the Amick Company. What physician can lend himself to assist a man who transgresses the highest laws of their guild—a man who not only holds a secret nostrum, but who asks that honest practitioners shall accept from him (for cash) a remedy of which they know nothing, guaranteed to do impossibilities!

"As this new aspirant for the shekels of the credulous is being 'pushed' and advertised even in some of our medical journals—we only ask our readers to notice the claims which this secret nostrum vender makes, and to remember that in not one of his cases of so-called tuberculosis has the existence of the tubercle bacilli been recorded. We can not blame a drowning man for catching at a straw, but what shall we say of a physician who guarantees that the straw he sells to each victim is a life-boat?

"Meanwhile Amick and Keeley form companies, and the companies make money, while the physicians are requested to become the willing agents in reaching and securing the misguided sufferers."

